# Simulator of the Human Intestinal Microbial Ecosystem (SHIME^®^): Current Developments, Applications, and Future Prospects

**DOI:** 10.3390/ph17121639

**Published:** 2024-12-06

**Authors:** Wei Zhu, Xiaoyong Zhang, Dong Wang, Qinghua Yao, Guang-Lei Ma, Xiaohui Fan

**Affiliations:** 1 College of Pharmaceutical Sciences, Zhejiang University, Hangzhou 310058, China; 12319103@zju.edu.cn (W.Z.); guangleima@zju.edu.cn (G.-L.M.); 2 Hangzhou Institute of Medicine, Chinese Academy of Sciences, Hangzhou 310000, China; shanezxy@163.com; 3 Department of Orthopaedics, Hangzhou TCM Hospital Affiliated to Zhejiang Chinese Medical University, Hangzhou 310007, China; hz-wdong@alu.zcmu.edu.cn; 4 The Second Clinical Medical College, Zhejiang Chinese Medical University, Hangzhou 310005, China; 20234052@zcmu.edu.cn; 5 Future Health Laboratory, Innovation Center of Yangtze River Delta, Zhejiang University, Jiaxing 314102, China; 6 The Joint-Laboratory of Clinical Multi-Omics Research Between Zhejiang University and Ningbo Municipal Hospital of TCM, Ningbo Municipal Hospital of TCM, Ningbo 315010, China

**Keywords:** simulator of the human intestinal microbial ecosystem (SHIME), human gastrointestinal microbiota, gut microbiota, application, food and nutritional science, drug development, gut health

## Abstract

The human gastrointestinal microbiota plays a vital role in maintaining host health and preventing diseases, prompting the creation of simulators to replicate this intricate system. The Simulator of the Human Intestinal Microbial Ecosystem (SHIME^®^), a multicompartment dynamic simulator, has emerged as a pivotal in vitro model for studying the interactions and interferences within the human gut microbiota. The continuous and real-time monitoring hallmarks, along with the programmatically flexible setup, bestow SHIME^®^ with the ability to mimic the entire human intestinal ecosystem with high dynamics and stability, allowing the evaluation of various treatments on the bowel microbiota in a controlled environment. This review outlines recent developments in SHIME^®^ systems, including the M-SHIME^®^, Twin-SHIME^®^, Triple-SHIME^®^, and Toddle SHIME^®^ models, highlighting their applications in the fields of food and nutritional science, drug development, gut health research, and traditional Chinese medicine. Additionally, the prospect of SHIME^®^ integrating with other advanced technologies is also discussed. The findings underscore the versatility of SHIME^®^ technology, demonstrating its significant contributions to current gut ecosystem research and its potential for future innovation in microbiome-related fields.

## 1. Introduction

The human gastrointestinal tract consists of distinct regions, including the stomach, small intestine, and colon, each comprising a unique anatomy, physiology, and microenvironment [1,2]. This intricate gut ecosystem hosts trillions of microbes collectively known as the gut microbiota, with bacteria being the most abundant and prominent microbes [3,4]. The gut microbiota significantly impacts human health by metabolising undigested nutrients, producing beneficial molecules, preventing pathogen colonisation, and training the immune system [5,6,7,8]. While the composition of the gut microbiota remains generally stable, its dramatic change in response to unhealthy factors may lead to various mental and physical diseases [9,10,11]. To better understand how the varying gastrointestinal environment contributes to health outcomes, in vivo studies are crucial. However, such studies often pose challenges due to ethical concerns, limited accessibility to intestinal samples, and high costs [12,13]. Moreover, the inherent complexity and variability among individuals often result in non-uniform data [14]. Hence, the development of robust in vitro models of the ileal microbiota has become vital.

**Figure 1:**
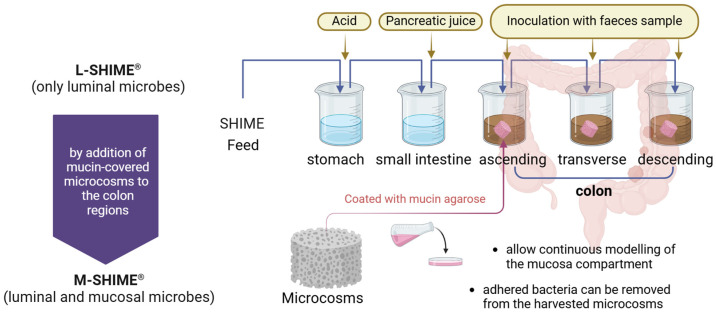
Schematic overview of the SHIME^®^ system, a dynamic in vitro model of the human gastrointestinal tract composed of several double-jacketed vessels that simulate the stomach, small intestine, and three main colon regions. All vessels are kept at 37 °C and anaerobic conditions by flushing the headspace of each compartment daily with N_2_ gas or N_2_/CO_2_ (9:1) gas mixture. The colon units in SHIME^®^ consist of the conventional setup that only harbours luminal microbes, and hence, it is also referred to as L-SHIME^®^, whereas the M-SHIME^®^ model was modified by incorporating a mucosal compartment that contains mucin-covered microcosms.

In vitro fermentation models designed to simulate various sections of the human gastrointestinal tract enable the investigation of microbial communities with programmable control over physiological parameters, such as nutrient availability and pH levels [15,16]. A series of systems with varying complexity have been developed and implemented in the field of gut microbiota research [17,18,19]. These simulated models can be classified as dynamic and static, with the dynamic models more closely reflecting the inherent condition of the human gut environment. Notable examples of dynamic models include the Simulator of Human Intestinal Microbial Ecosystem (SHIME^®^) developed in 1993 as one of the first systems capable of maintaining both small and large intestinal microbial communities through continuous fermentation [20,21]. The TNO Gastro-Intestinal Model (TIM-1 and -2) was designed to simulate dynamic stomach and small intestinal conditions with a special setup that incorporates nutrient absorption via dialysis [21]. Similarly, the Dynamic Gastric Model (DGM) introduced in 2007 mimics human gastric processing with high accuracy including gastric mixing, shear rates, and peristalsis [22]. Another dynamic system, the Human Gastric Simulator (HGS) model, was introduced by incorporating antral contractions and controlled gastric emptying in a continuous manner [23]. The SIMulator of the GastroIntestinal tract system (SIMGI) offers the flexibility to simulate gastric and colonic fermentative processes either jointly or separately [24]. In addition, Wiese et al. introduced the Copenhagen MiniGut (CoMiniGut), a high-throughput miniaturisation for small-volume experiments, facilitating studies on rare and expensive bio-actives [25]. Among these dynamic advanced simulators, SHIME^®^ is among the most extensively used and validated in vitro dynamic models, as it features a continuous and real-time monitoring setup and entire gastrointestinal tract integration, which is particularly useful for colon-specific studies (Figure 1), making it the primary focus of this review [20,26].

While the number of reviews focusing on in vitro gut models has been increasing, SHIME^®^ is typically discussed only briefly as one technique among the models. To the best of our knowledge, there are only two reviews specifically centred on the SHIME^®^ system. The first, presented as a book chapter in 2015, introduces the general features, protocol, and outputs of SHIME^®^, with the applications remaining to be discussed [20]. The second paper focused on SHIME’s application in studying the mode-of-action of probiotics in the gastrointestinal tract [27]. Hence, this review will outline the recent key developments in SHIME^®^, highlighting its ability to accurately mimic the human gastrointestinal environment and its diverse applications in food and nutritional science, drug development, gut health research, and traditional Chinese medicine. We will begin by providing a general overview of the SHIME^®^ technology, including its history, working principles, and versional developments. Next, we will discuss the current status and practical applications of SHIME^®^ in various research contexts. Finally, we will explore future trends and challenges facing the SHIME^®^ platform and further consider how ongoing advancements may enhance its effectiveness and accuracy in appreciating gut microbiota interactions and the implications for human health.

## 2. Overview of SHIME^®^

SHIME, an acronym for the Simulator of the Human Intestinal Microbial Ecosystem, has been a widely used tool for studying gut microbiota dynamics [20]. In 2010, the name SHIME^®^ was jointly registered by ProDigest and Ghent University, marking a formal collaboration to further advance this technology. Such a simulator allows for the cultivation of a complex intestinal microbial ecosystem over an extended period while maintaining representative conditions of different intestinal regions, to closely mimic in vivo gut conditions [28].

### 2.1. History of SHIME^®^

SHIME^®^ is a multicompartment dynamic simulator of the human gut, originally developed in 1993 by Verstraete and co-workers from Ghent University [20]. The creation of the model was driven by the realisation that the faecal microbiota, often used as a proxy for gut microbiota research, significantly differs from the in vivo colon microbiota in terms of community composition and metabolic activity. Initial efforts to mimic the colon environment, such as the inoculation of faecal microbiota into single-stage chemostats, were only effective for short periods [28,29]. This limitation arose because key environmental parameters such as the pH, redox potential, available nutrients, and microbial population dynamics change constantly within the reactors. In 1981, Miller and Wolin collectively addressed some of these challenges by developing a semi-continuous fermenter, which could simulate the intermittent replenishment of nutrient media, allowing for a more sustained microbial environment [30]. However, the complexity of the colon, together with its varying regions and unique conditions, makes it challenging to simulate a representative culture of the colon microbiota in a single compartment. To overcome the limitations of single-compartment models, Macfarlane et al. introduced a multi-compartment reactor system that could simulate the distinct conditions of different regions of the colon [31]. Upon these advancements, SHIME^®^ was developed as one of the most representative gut simulators of this generation [17,32]. Technically, SHIME^®^ is an evolution of the gut simulator introduced by Macfarlane from the University of Reading. While the original Reading model provided valuable insights into the colonic microbiota, SHIME differentiates it by incorporating the conditions of the upper digestive tract (stomach, small intestine), thereby offering a more comprehensive simulation of the entire gastrointestinal system [20,28].

### 2.2. The Modules and Working Principles of SHIME^®^

SHIME^®^ generally consists of a succession of five compartments that replicate different sections of the digestive tract (Figure 1). It contains double-jacketed glass vessels that are connected through peristaltic pumps. The first two compartments simulate the upper digestive tract (stomach and small intestine), where factors such as the acidity, digestive enzymes, and nutrient absorption play significant roles. These compartments are crucial for creating the appropriate conditions for downstream microbial colonisation and fermentation in the colon. The next three compartments simulate the lower digestive tract (the ascending, transverse, and descending colon), where the gut microbiota is most active and the inoculation of faeces samples occurs. In these compartments, SHIME accurately mimics the pH gradients, nutrient availability, and anaerobic conditions in the real human colon, which allows for the investigation of region-specific microbial dynamics, including fermentation processes and the production of metabolites such as short-chain fatty acids [20,28]. Beyond the commonly described configuration with five bioreactors, there are numerous alternative SHIME^®^ setups tailored to specific research needs. These include the one- or two-colon compartment model, which simplifies the system by representing only the proximal and/or distal colon to focus on key regional differences in microbial activity and metabolism [29,33]. Another example is the single stomach bioreactor feeding multiple colon lines, enabling comparative studies of microbial responses to identical upstream conditions across different ecosystems [34,35,36]. Additionally, specialised configurations can be designed to study specific aspects of gut physiology such as pH-specific impacts or to incorporate mucosal simulators and biofilms to explore host–microbe interactions [22,37]. Furthermore, dynamic modular setups allow for the exclusion of specific compartments based on research objectives such as isolating colonic processes from upstream digestive steps [38].

A typical SHIME experiment consists of four stages, each meticulously designed to evaluate the dynamics of the gastrointestinal microbial community under controlled conditions [28]. The initial stabilisation period (normally 2 weeks) allows the microbial community to adapt to the specific environmental conditions established in the respective colon regions. This adaptation is crucial, as it allows for the stabilisation of microbial populations to provide a balanced ecosystem for the subsequent measurements. For the second basal period (~2 weeks), the reactor operates under nominal conditions to establish a stable baseline representative of normal gut microbiota activity. Key parameters such as the microbial composition, pH level (monitored during both the stabilisation and basal periods), and other metabolic activities are measured. These data serve as a vital reference point for evaluating the effects of any interventions introduced later.

In the third stage (around 2–4 weeks), a specific treatment (e.g., probiotic, prebiotic, or bioactive molecules) is introduced to assess the impact on the gastrointestinal microbial community. The final stage of washout (2 weeks or longer) aims to assess how long the effects induced by the tested substance persist in its absence, with the duration varying according to the specific aims of the experiments. This washout period is essential for understanding the stability and longevity of any changes observed in the microbial community following the treatment [28].

### 2.3. Parameter Setup of SHIME^®^

SHIME’s stomach and small intestine modules follow a fill-and-draw principle, wherein a defined nutritional medium is added to the gastric compartment three times a day, along with pancreatic and bile liquid to the small intestine compartment. Following the digestion process in the gastric and intestine compartments, the resulting slurry is pumped into the ascending colon vessel, initiating colon digestion. The three colon compartments are continuously stirred to ensure thorough mixing with constant volume and pH control to simulate natural digestive conditions. The system’s retention times, which represent the time it takes for the medium to move through the modules, can be adjusted by altering the flow rates from the gastric and small intestine or by changing the volumes of the colon. The retention times can vary from 24 h to 72 h, depending on the human target group being simulated (e.g., individuals with different health conditions). To promote the growth of diverse microbial communities, a specifically optimised gut microbiota medium is often required as no such universal medium is fit to culture all the gut microorganisms. The composition of a culture medium for microbiological studies is typically composed of water, a carbon source, a nitrogen source, and some mineral salts [39]. The temperature is typically set to physiological levels (about 37 °C) to simulate human body temperature. Nutrient availability is controlled through the addition of specific growth media tailored for each stage of digestion, introduced at defined intervals [20].

**Figure 2:**
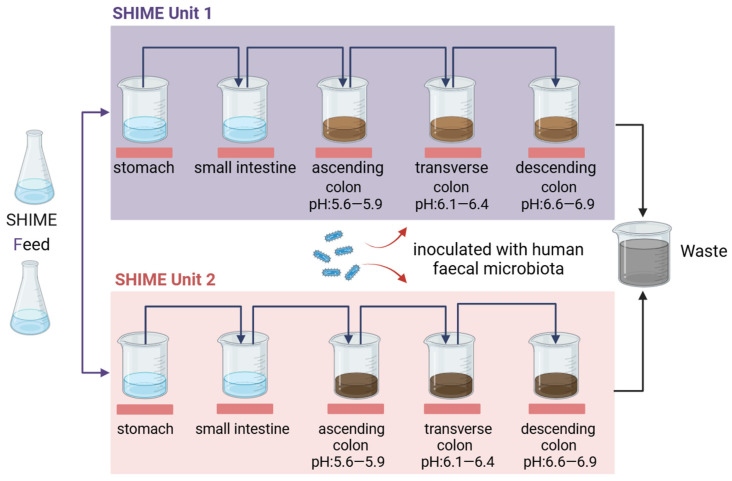
Diagrammatic representation of the Twin-SHIME^®^ model. The model consists of two identical SHIME units (units 1 and 2) that allow researchers to directly compare the effects of different treatments or environmental conditions on gut microbial communities in parallel. The inoculum for faecal microbiota is only introduced into the colon bioreactors.

Additionally, the gastric compartment was typically maintained at a fixed pH of 2.0, but with the introduction of a fully computer-controlled SHIME system, specific pH profiles can now be established to more accurately replicate the dynamic changes in pH during digestion in both gastric and intestine modules. While the small intestine typically operates at slightly acidic to neutral pH conditions, the pH in the colon compartments is maintained within specific ranges, that is between 5.6 and 5.9 in the ascending colon, 6.1–6.4 in the transverse colon, and 6.6–6.9 in the descending colon (Figure 2) [40]. The pH gradient closely simulates the conditions found in the real human colon and is critical for understanding the impact of microbial activity in different gut regions. Mixing of the digestive slurry in each compartment is achieved using magnetic stir bars to ensure homogeneous conditions throughout the compartments. The entire SHIME system is kept in anaerobic conditions by flushing the headspace of each compartment daily with N_2_ gas or a N_2_/CO_2_ (9:1) gas mixture, mimicking the low-oxygen environment of the gastrointestinal tract [41]. By carefully setting these parameters, SHIME^®^ provides a robust platform for studying the complex interactions within the gut microbiota and their implications for human health and disease.

### 2.4. Development of SHIME^®^

Currently, the SHIME^®^ model is employed to investigate the physical, chemical, enzymatic, and microbiological parameters of the human gut under controlled conditions. Researchers have optimised and adapted the SHIME^®^ (ProDigest, Ghent, Belgium) model for various experimental purposes, resulting in specialised versions such as M-SHIME^®^ [37], Twin-SHIME^®^ [34], and Triple-SHIME^®^ (Table 1) [35]. The conventional SHIME^®^ focuses on observing luminal microorganisms; hence, it is also referred to as L-SHIME^®^ [20]. Yet, the human gut harbours a vast and intricate microbial community that plays a crucial role in maintaining health by preventing pathogen colonisation and producing essential nutrients, and these microorganisms are not randomly distributed in the intestinal tract. Those adhering to the mucosa of the intestinal wall are particularly significant, as they act as a “barrier” to influence the mucosal immune response and occupy specific ecological niches [42].

To address this concern, M-SHIME^®^ (i.e., Mucosal-SHIME^®^) was developed by augmenting L-SHIME^®^ with microstructures that mimic the mucin cap across the three colonic regions (Figure 1) [37]. The innovative setup allows bacteria adhering to the mucosal layer to colonise these structures, forming a mucosal microcosm within the reactor. By replacing half of this mucosal microecology approximately every three days, researchers can effectively simulate the natural renewal process of the intestinal mucosal layer, enabling the continuous modelling of mucosal dynamics over time. Furthermore, the model permits the harvesting of adherent bacteria from the replaced mucosal microcosm, facilitating the detailed characterisation of the mucosal community. This capability is crucial for investigating the specific interactions between the mucosal-localised microbiota and host epithelial cells, which are essential for maintaining gut health and immune function [43].

The Twin-SHIME^®^ system (Figure 2), developed by Van den Abbeele and co-workers, innovatively connects two SHIME^®^ systems in parallel, facilitating liquid transfer between reactors while maintaining identical pH and temperature conditions through a double-ended pump [34]. This configuration is particularly advantageous for simultaneous studies of different interventions on the gut microbiota, ensuring consistent environmental conditions across both systems and enhancing the reliability of experimental outcomes. When focusing on a single colonic region, it is feasible to set up several parallel SHIME^®^ units, allowing for a comprehensive analysis of microbial dynamics. However, when the area of interest spans three colonic regions, Twin-SHIME^®^ limits the capacity for handling repetitions, making Triple-SHIME^®^ a more suitable solution. Unlike Twin-SHIME^®^, which often permits the simulation of the stomach and small intestine within a single compartment, Triple-SHIME^®^ necessitates separate compartments for the gastrointestinal tract [35,36]. This separation enhances the simulation’s fidelity, accommodating the unique physiological characteristics of each digestive segment.

Furthermore, Bondue et al. adapted the SHIME^®^ model to study microbiota in young children, replicating their unique digestive processes and colonic environments [44]. This modified model (i.e., Toddle SHIME^®^) is particularly effective for assessing the effects of prebiotics and probiotics on young children’s microbiota, as well as for evaluating the metabolism of drugs and endocrine disruptors that may affect their health. The Mini-Colon Model (MiCoMo), a benchtop multi-bioreactor system that consists of triplicate bioreactors working independently, has gained popularity in laboratory settings due to its high throughput and multiplexing capabilities, providing cost-effective and superior simulation outcomes for gut microbiota research [45]. To further enhance the SHIME^®^ model, Vasquez et al. integrated a chemical gas sensor utilising carbon nanotubes for continuous monitoring of gaseous biomarkers within the simulator [46]. This advancement establishes a comprehensive sensor platform that facilitates real-time monitoring of gaseous biomarkers in a cost-effective manner, improving the model’s utility for studying the gut microbiota and their metabolic activities. Through these innovations and adaptations, the SHIME^®^ model is continuing to be a vital tool in understanding gut microbiota interactions and their implications for human health.

### 2.5. Advantages and Limitations of SHIME^®^

SHIME^®^ offers unique advantages, making it a valuable tool in gut microbiota research. Table 2 provides a brief summary of the advantages and limitations of the SHIME^®^ technology [20,28]. One of the primary strengths is its multicompartment design, which simulates and integrates the entire gastrointestinal tract, allowing for a more accurate representation of microbial dynamics across the stomach, small intestine, and the colon. The real-time monitoring setup enables the continuous measurement of various parameters, such as pH, temperature, and gas production, providing detailed insights into microbial activity. Additionally, SHIME’s long-term stability and controlled conditions facilitate extended experimental periods, allowing researchers to manipulate defined environmental factors such as nutrient availability and fermentation conditions. The model can also be modified for specific research needs, with adaptations such as M-SHIME^®^ for studying mucosal interactions and Twin- or Triple-SHIME for conducting simultaneous parallel studies across multiple compartments. This flexibility enhances its applicability in various research contexts.

As for the limitations of the SHIME^®^ system, one significant drawback is that the absorption unit employs mainly semi-permeable membranes, which limits its ability to accurately simulate active absorption processes. Furthermore, the entire gastrointestinal simulation system lacks a bionic peristaltic mechanism, resulting in a less realistic representation of gut motility and its effects on microbial dynamics and nutrient transport. Meanwhile, the absence of host cells within the system makes it challenging to replicate crucial interactions such as those between the microbiota and epithelial cells. Consequently, while SHIME^®^ provides valuable insights into gut microbiota research, these limitations highlight the need for complementary methods to better understand the multifaceted hallmarks of the human gut ecosystem.

Overall, the SHIME^®^ model provides a controlled, reproducible in vitro system to study gut microbiota interactions with food/nutrition components, pharmaceuticals, and microbial interventions. However, it is not designed to replace in vivo trials but rather to complement them. SHIME^®^ serves as a valuable preliminary screening tool for optimising formulations, reducing the time and financial burden associated with in vivo studies. Additionally, it supports ethical research practices by minimising the reliance on animal studies and offering practical benefits for initial assessments. While SHIME^®^ provides critical insights, it does not replicate the systemic and host-level responses in living organisms; thus, in vivo validation of the findings is often required to ensure a comprehensive understanding of gut microbiota-related interventions [17,47,48].

## 3. Applications of SHIME^®^

At present, SHIME^®^ has become a significant technological platform for investigating the effect of food and drugs on intestinal microecology. Researchers have leveraged this technology to study the metabolism of food bioactive substances, the dynamics of drug release, and the function and diversity of bacteria within the gut ecosystem. Its sophisticated design allows for real-time monitoring and flexible manipulation of the gastrointestinal conditions, making it an indispensable tool in the fields of food safety and nutrition science, pharmacology, and microbial ecology [47,49,50]. Furthermore, several comparative studies have substantially demonstrated that the SHIME^®^ findings closely align with in vivo results. For example, studies comparing SHIME^®^ with human faecal samples in vivo have demonstrated similar patterns in metabolite fermentation and enzymatic activity following dietary interventions or antibiotic treatments [32]. Another validation study by Duysburgh et al. also indicated that the in vitro gut model (M-SHIME^®^) and in vivo trial yielded consistent stimulatory effects of oat ingredients on lactobacilli, underscoring the power of the SHIME model to predict the in vivo response to dietary modulation [51]. Below, we will present key examples of SHIME^®^ technology applied across various research fields.

### 3.1. SHIME^®^ Application in Food and Nutrition Science

SHIME^®^ technology is extensively utilised in the field of food safety and nutrition research. Its principal applications include the simulation of food digestion and absorption processes, investigation of nutrient metabolic pathways, and the assessment of their impact on the intestinal microbiota. The model allows for the examination of the production and role of metabolites such as short-chain fatty acids (SCFAs), primarily butyrate, acetate, and propionate, which are crucial metabolites produced by the gut microbiota and play various important roles in gut health [52]. Among these, butyrate is especially important for maintaining the integrity of the gut barrier as an energy source for intestinal epithelial cells. Several studies have utilised the SHIME^®^ model to investigate the effects of different food additives, fibres, and probiotics on the gut microbiota and their metabolic outcomes [53,54]. For instance, Gonza et al. employed the SHIME^®^ model to investigate the effects of several food additives, such as polysorbate 80, sucralose, maltodextrin, and sodium nitrite, on the gut microbiota and metabolic activity of individuals under healthy and unhealthy conditions. They found that polysorbate 80 and sucralose reduced butyrate-producing bacteria (e.g., *Roseburia*, *Faecalibacterium prausnitzii*) while increasing bacterial species such as *Enterococcus* and *Veillonella*, which are positively correlated with intestinal inflammation and fibrosis [55].

Van den Abbeele et al. demonstrated that the intake of long-chain arabinoxylan and inulin significantly increased the production of propionate and butyrate in the gut. The study further showed that *Bifidobacterium longum* was stimulated by long-chain arabinoxylan, while *Bifidobacterium adolescentis* responded to inulin intake [56]. Similarly, Tails Fernanda et al. explored the effect of fermented milk with fruit pulp on the in vitro intestinal microbiota using the SHIME^®^ model. They reported that this probiotic product increased the production of beneficial SCFAs while reducing harmful ammonium ions, indicating its potential to promote gut health [57]. A related study by Herkenhoff et al. showed that fermented milk formulations, both with and without cashew by-products, had a positive impact on the intestinal microbiota using the SHIME^®^ system. The inclusion of cashew by-products led to significant changes in the proportions of specific microbial groups and metabolite production, notably increasing the abundance of *Bifidobacterium* spp., *Lactobacillus* spp., *Actinobacteria*, and *Lacticaseibacillus paracasei* F-19, along with elevated levels of phenolic compounds, ammonium, and SCFAs [58].

SHIME^®^ technology is also valuable for assessing the digestion and metabolism of nutrients in the gastrointestinal system, particularly in the presence of intestinal microbiota. Liu and co-workers employed SHIME^®^ to investigate the digestion and bioavailability of two soybean components: polysaccharides and isoflavones [59,60]. Their findings revealed that both polysaccharides and isoflavones were primarily broken down and utilised by the gut microbiota in the colon. Moreover, soybean polysaccharides can promote the growth of beneficial probiotics and enhance the ability to inhibit pathogenic bacteria, underscoring their potential role as a functional food to improve the gut microbiota. SHIME^®^ is also applied to evaluate the influence of the human gastrointestinal tract on the metabolism of endocrine disruptors in food, such as bisphenol A (BPA), a chemical widely used in making food plastic containers. Wang and co-workers demonstrated that BPA exposure significantly altered the microbial community in colons during digestion, with the BPA-degradable bacteria (e.g., *Microbacterium*, *Alcaligenes*) being up-regulated [61]. Another disrupting chemical, chlorpyrifos (CPFs), an insecticide commonly used on fruit and vegetable crops, was reported to induce noticeable dysbiosis in the gut microbial community. In particular, CPFs led to the proliferation of *Bacteroides* spp. and *Enterococcus* spp., alongside a reduction in beneficial strains such as *Bifidobacterium* spp. and *Lactobacillus* spp. [62].

### 3.2. SHIME^®^ Application in Drug Development

Given the functional characteristics, SHIME^®^ technology is also adept at simulating the absorption, release, and metabolism of drugs in the gut and can be used to investigate the interactions between gut microorganisms and pharmaceutical compounds [38,50]. This capacity makes SHIME^®^ an important platform for drug development. Tabare et al. utilised SHIME^®^ technology to evaluate the release profile of Eudragit^®^ FS microparticles that contain bacteriophage LUZ19, aiming to develop phage oral dosage forms for targeting bacteria in the colon [63]. Derave and co-workers employed SHIME^®^ to evaluate the magnesium bioavailability of 15 commercial magnesium supplement formulations through dissolution assays. The findings demonstrated a wide variation in absorption and dissolution among the magnesium products, providing a valid in vitro SHIME-based methodology for predicting the in vivo bioavailability and effectiveness of micronutrients [64]. As for the studies on drug metabolism, Grootaert et al. utilised the Twin-SHIME^®^ system to explore the prebiotic effects of aglycone-based xyloglucan oligosaccharides and insulin, revealing that distinct degradation patterns occurred in the different regions of the colon and demonstrating the potential of this differentiation to deeply understand specific drug metabolism and reaction conditions [65]. Similarly, Martínez-López et al. assessed the degradation of insulin microcapsules prepared by enzymatic gelation of arabinoxylan using SHIME^®^ and found that arabinoxylans microcapsules can inhibit the simulated conditions of the upper gut system and act as a barrier for insulin delivery to the colon [66].

SHIME^®^ has also proven valuable in developing complementary therapeutic strategies to address the chemotherapy- or antibiotic-induced gut dysbiosis. Ichim and co-workers employed the SHIME^®^ model to evaluate a formulation combining a probiotic with digestive enzymes, demonstrating its capacity to mitigate chemotherapy-induced gut dysbiosis. Their findings underscored the formulation’s potential as a supportive strategy to preserve gut health during cancer treatment [67]. Similarly, Calatayud et al. showed that long-term lactulose administration significantly reduced the growth of viable *Clostridioides difficile* cells following clindamycin treatment using the SHIME^®^ platform. This intervention also mitigated antibiotic-induced dysbiosis by reshaping the microbial community, stimulating the production of health-promoting metabolites, and exhibiting a notable bifidogenic effect, highlighting its potential as a therapeutic strategy to counteract antibiotic-associated disturbances and recover the gut microenvironment [68].

Additionally, the integration of SHIME^®^ technology also provides extensive data that inform the study of drug combination and delivery strategies. Li et al. employed the SHIME^®^ model to examine the in vitro effects of a combination of colistin and amoxicillin on the intestinal microbiota and its antibiotic resistance, as well as to evaluate the recovery of the gut microbiota upon faecal microbiota transplantation (FMT) treatment [69]. The study highlights the potential of the administration of antibiotics and application of FMT in clinical settings. Moreover, Mccoubrey et al. leveraged SHIME^®^ to compare the efficacy of two colonic delivery strategies for ulcerative colitis (UC) by measuring the release of mesalazine from both prodrug-mediated and formulation-mediated approaches. The findings underscored that the approach selected for colonic drug delivery could significantly impact the effectiveness of UC treatment [70]. Another relevant example is that of Cesar et al., who investigated the effects of a citrus flavonoid mixture combined with metformin for treating pre-diabetes; the results demonstrated the potential of combination administration to lower blood glucose levels and enhance glucagon-like peptide-1 (GLP-1) in pre-diabetic patients [71]. Additionally, Jannin and co-workers utilised a modified SHIME^®^ model to simulate the gastrointestinal tract conditions of patients with exocrine pancreatic insufficiency (EPI) to evaluate the dissolution characteristics and enzymatic activity of five pancrelipase-filled capsules. The study demonstrated that the Next Generation Enteric (NGE) capsule, containing a simplified powder formulation of pancrelipase, achieved a comparable release profile, enzyme protection, and digestive efficiency to Creon^®^, the market reference capsule with a more complex formulation, underscoring the potential of NGE capsules as a cost-effective alternative for treating EPI [72]. Upon these applications, SHIME^®^ technology has provided critical insights into drug metabolism, efficacy, and the complex interactions between drugs and the gut microbiota.

### 3.3. SHIME^®^ Application in Gut Health Research

The gut microbiota significantly impacts human health and physiology, playing a vital role in establishing the mucosal barrier and maintenance of gut homeostasis. These gut microbes influence the intestinal barrier by regulating the expression and distribution of tight junction proteins, as well as modulating mucus secretion and mucin glycosylation [73]. The study by Piche et al. indicated that an impaired gut barrier was a critical factor in the predisposition to intestinal diseases, such as irritable bowel syndrome (IBS), and the results demonstrated that mucosal soluble mediators can reproduce functional permeability changes and molecular alterations, including zonula occludens-1 (ZO-1) mRNA expression, as observed in patients with IBS [74]. Suligoj and co-workers utilised the SHIME model, along with the Caco2 cell line and gut organisms, to investigate the adult gut microbiota and gut barrier function. They demonstrated that human milk oligosaccharides (HMOs) are able to shape the infant gut microbiota by selectively stimulating the growth of *bifidobacteria* and also have the ability to modulate immune function and enhance gut barrier integrity, indicating that HMOs may confer health benefits even in adults [75].

Importantly, investigating the gut microbiota could serve as an alternative strategy for preventing clinical disease. Salgaco et al. examined the effect of infant cereals containing *Bifidobacterium animalis* ssp. *lactis* BB-12^®^ on the gut microbiota of infants using SHIME^®^. After inoculating the faecal microbiota of three young children, those consuming BB-12^®^ cereals experienced beneficial growth of *Lactobacillus gasseri*, linked to a reduced risk of allergic rhinitis in children, and *L. kefiri*, associated with obesity prevention [76]. In another study, Marzorati et al. employed SHIME^®^ for the first time to assess the effect of the spore-based oral probiotic MegaSporeBiotic™ on gut microbial activity and community composition, highlighting the potential of probiotic treatment to alter gut metabolism and increase bacterial diversity [77]. Psychobiotics are probiotics that influence central nervous system functions through gut–brain axis modulation. De Oliveira et al., using the SHIME^®^ model, demonstrated that the combination of *Lactobacillus helveticus* R0052 and *Bifidobacterium longum* R0175 positively influenced gut microbiota composition. This combination increased γ-aminobutyric acid and SCFA levels, reduced pro-inflammatory cytokines, and elevated anti-inflammatory cytokines, suggesting that the probiotic blend holds promise as an adjunctive treatment for anxiety [78].

Additionally, existing studies have employed the M-SHIME^®^ model to investigate the inhibition of invasive *Escherichia coli* in mucus by specific probiotics and prebiotics, such as *Limosilactobacillus reuteri* 1063 and arabinoxylans. The interventions help limit the adverse effects of pathogens on the host by reducing their proximity to epithelial cells, which in turn minimises the potential for infection and inflammation [56]. Lambrecht et al. used the M-SHIME^®^ model to evaluate the transfer of a resistance plasmid containing multiple antibiotic resistance genes from commensal *Escherichia coli* to the human gut microbiota. This study highlighted the risks of horizontal gene transfer, which can promote the spread of antibiotic resistance within the gut, posing a significant challenge for treatment options in humans [79].

### 3.4. SHIME^®^ Application in Traditional Chinese Medicine

The study of the metabolic process of Chinese herbs in the digestive tract is crucial to reveal medicinal substances and their intrinsic therapeutic mechanisms. Currently, research on the efficacy of traditional Chinese medicine largely relies on animal experiments, but the results cannot be directly applied to humans due to species-specific differences. Additionally, the complexity of traditional Chinese medicine compositions makes it challenging to uncover the exact metabolic process in vivo. Therefore, constructing an in vitro bionic digestive system for traditional Chinese medicine can provide a more effective approach to discovering their mechanism of action.

Currently, SHIME^®^ technology is still in the exploratory stage within the realm of Chinese medicine research. Wu and co-workers combined SHIME^®^ technology with co-culture techniques involving the intestinal microbiota and endothelial cells to investigate the anti-inflammatory effects of black heather (*Aronia melanocarpa*) polyphenols and their modulation of the intestinal microbiota, and they found that the aronia polyphenols modulated the gut microbial composition by inducing beneficial SCFA production and preventing inflammatory stress of endothelial cells [80]. Similarly, Liu et al. utilised the SHIME^®^ model to reveal how orange-peel soup modulates the intestinal microbiota and short-chain fatty acid metabolites, offering insights into its potential clinical application for treating vomiting and eructation [81]. Yao and co-workers also made a significant discovery using SHIME^®^, proving that the classical Chinese medicine preparation Chaihu Shugan San can significantly improve the intestinal mucosal barrier function of individuals with liver qi stagnation syndrome, along with potent immunomodulatory effects on intestinal tumours [82]. This provides new perspectives for exploring the mechanism of traditional Chinese medicines on gut microbial ecosystems, particularly for some chronic diseases (e.g., obesity, diabetes, and cardiovascular disease). For diabetes, Yang et al. demonstrated that the long-term oral administration of the aqueous extract of *Lonicera japonica* flos (LJE) can significantly alter the composition and structure of the faecal microbiota in individuals with type 2 diabetes (T2D). Using the SHIME^®^ system, they showed that LJE promoted short-chain fatty acid (SCFA) production, which may contribute to its therapeutic effects on T2D and enhance the efficacy of TCM for metabolic disorders [83]. Another specific example is Chenpi, a well-known Chinese medicine that has been clinically used for cholesterol reduction and various gastrointestinal symptoms. Maria Falduto et al. employed the SHIME^®^ model to examine how the microbial community changes during treatment with two Chenpi extracts, thereby revealing the anti-obesity effects of Chenpi extracts rich in polymethoxyflavonoids and laying the groundwork for the future identification of obesity-related biomarkers within the gut microbiota [84].

## 4. Future Trends and Challenges of SHIME^®^

Generally, SHIME^®^ systems lack feedback mechanisms from the host, making the development of sophisticated host–microbe interactions essential; thus, an intriguing tendency for SHIME^®^ is the integration of continuous dynamic models with additional host cells to enhance its relevant nature to the real gut environment. In fact, studies have been conducted to explore the effect of samples from in vitro models on Caco2 cells and the immune cells THP-1 and U937, to study the adherence, cytokine production, or gene expression [48,85]. In the future, expanding the host cell types (e.g., enterocytes, goblet, and Paneth cells) within the SHIME^®^ system, along with incorporating innovative technologies such as omics (e.g., genomics, metabolomics), will provide deeper insights into gut microbe–host interactions and related metabolic pathways.

Recently, organoids have emerged as a promising 3D culture system, bridging the gap between 2D and in vivo models. These multicellular structures can recapitulate the complex architecture and cellular interactions found in real tissues, overcoming the limitations of reductionist in vitro models [86]. Intestinal organoids hence offer a valuable alternative for modelling the gastrointestinal tract and for exploring the interactions between the gut microbiota and human epithelium. Organoids are frequently derived from adult stem cells isolated from the small intestine or the colonic epithelium, giving rise to sophisticated constructs known as enteroids or colonoids [87]. However, challenges remain in effectively combining the organoids with a microbiota, as trapping microbes within organoids has proven difficult. Another related advancement is the organ-on-chip model of the colon, which represents a breakthrough in studying the human colonic mucosa. This model can produce a thick mucous layer with a bilayer structure that closely mirrors in vivo observations, enabling more precise studies of microbial interactions and real-time responses within the SHIME^®^ system [88]. The development of computational ‘‘in silico” models that integrate artificial intelligence and machine learning techniques also represents a future direction for SHIME^®^. These technologies, along with digital twin systems, will improve the design and optimisation of in vitro systems and provide a smarter and faster evaluation of SHIME^®^ models. Moreover, the growing interest in personalised medicine can leverage SHIME^®^ technology to model individual variations, paving the way for tailored treatments based on unique microbial profiles [89].

While the SHIME^®^ system has provided dramatic insights into the human gut microbiota, it is crucial to acknowledge the inherent limitations of in vitro simulators. The complexity of the human gut environment is challenging to fully replicate, making it essential to continually refine SHIME^®^ models to better reflect actual gut conditions. Additionally, standardising methodologies across different studies is also necessary to facilitate comparison and reproducibility, ensuring that SHIME^®^ continues to be a valuable tool in microbiota research.

## 5. Conclusions

Overall, SHIME^®^ is an exceptional tool for researchers seeking to delve into the intricacies of the gut microbiota and their interference on human health. Its ability to replicate the human gut environment with high accuracy makes it invaluable for studying the intestinal microbial dynamics across the fields of food and nutritional science, pharmacology, and gut ecosystem. Despite the inherent limitations and technical challenges associated with in vitro models, the insights gained from SHIME^®^ into gastrointestinal processes are significant and impactful. As research advances, the integration of SHIME^®^ with advanced technologies, such as omics, organoid and organ-on-chip systems, or potential personalised medicines, promises to enhance its relevance in microbiota research and clinical applications.

## Figures and Tables

**Table 1:** Development of SHIME^®^ models and the key hallmarks.

Model	Incorporated Modules	Key Hallmarks
L-SHIME^®^ (conventional SHIME^®^)	consists of five sequential compartments mimics the five different regions of the human intestinal tract.	observes luminal microbes under controlled conditions. assesses the dynamics of the human gastrointestinal microbiota
M-SHIME^®^	adds microstructures into L-SHIME^®^ system simulates the mucin caps of three colonic regions.	enables bacteria to adhere to the mucosal layer replicates the natural renewal process of the intestinal mucosal layer.
Twin-SHIME^®^	consists of two parallel SHIME^®^ systems each system contains the same compartments as L-SHIME^®^	each SHIME^®^ system can run independently enables comparative studies of different interventions on gut microbiota
Triple-SHIME^®^	contains three separate SHIME^®^ systems builds on the functionality of Twin-SHIME^®^	conducts more comprehensive comparative studies relative to Twin-SHIME^®^ each system can mimic distinct sections of the human gastrointestinal tract
Toddle SHIME^®^	contains the same five sequential compartments as L-SHIME^®^ faeces inoculated in the three colons were collected from a young child (1–2 years old)	facilitates the gut microbiota study of young children is adapted to evaluate prebiotic and probiotic impacts on the toddler microbiota

**Table 2:** Advantages and limitations of the SHIME^®^ system.

Advantages	multicompartment design replicates and integrates the entire gastrointestinal tract (i.e., stomach, small intestine, and colon) continuous and real-time monitoring allow for dynamic measurement of various parameters (e.g., pH, temperature, and gas) within the simulator programmatically controlled setup enables manipulation of defined environmental factors (e.g., nutrient availability, fermentation conditions) long-term stability ensures the maintenance of gut microbiota stability over an extended timeframe high adaptability and flexibility facilitate its evolution into advanced models such as Twin- and Triple-SHIME^®^ interindividual variability can be studied using this system variable transit and retention times
Limitations	the stabilisation period drives the microbiota to adapt to the system and away from the intestinal environment absence of a small intestinal microbiota use of semi-permeable membrane limits the accuracy of the simulated absorption processes lack of bionic peristaltic process restricts the model’s capacity to mimic gut motility absence of host cells, (e.g., epithelial, immune cells) in the SHIME^®^ system limits the study of microbe–host interactions current SHIME models have limited integration with advanced technologies such as AI, machine learning, and digital twin systems
